# A Review of Interstitial Pneumonitis Caused by Elotuzumab Through Case Discussions and Academic Writings

**DOI:** 10.7759/cureus.11162

**Published:** 2020-10-25

**Authors:** Ghulam Ghous, Hafiz Muhammad Hassan Shoukat, Zahid Ijaz Tarar, Muhammad Usman Zafar

**Affiliations:** 1 Internal Medicine, University of Missouri, Columbia, USA; 2 Internal Medicine, Premier Health/Wright State University, Dayton, USA; 3 Hospital Medicine, Lehigh Valley Health Network, Allentown, USA

**Keywords:** interstitial pneumonitis, elotuzumab, multiple myeloma

## Abstract

Patients with relapsed or refractory multiple myeloma have undergone two or three previous therapies are now being treated with a humanized IgG1 monoclonal antibody elotuzumab (HuLuc63) that targets F7 signalling lymphocytic activation molecule F7 (SLAMF7)- a signalling lymphocytic activation molecule. It is combined with dexamethasone and lenalidomide/pomalidomide for therapy. Adverse effects associated with elotuzumab consists of peripheral neuropathy, fever, constitutional symptoms (fatigue, headache, decreased appetite), and infections. A rare side effect of interstitial lung disease has only been observed in a single case. There are two case studies presented below of hypoxic respiratory failure upon this monoclonal antibody treatment both were successfully treated with steroid therapy. This article brings forth the hypothesis that elotuzumab can cause pneumonitis, and discontinuation of elotuzumab along with high-dose corticosteroids helps reverse the pneumonitis.

## Introduction

Humanized IgG1 monoclonal antibody elotuzumab (HuLuc63) that targets signalling lymphocytic activation molecule F7 (SLAMF7) [1-3]. Multiple myeloma cell death is caused by it through direct activation of natural killer cells targeted at neoplastic plasma cells through Fc receptors (CD16), SLAMF7 pathway and by tagging them for recognition of ADCC (antibody-dependent cellular toxicity). With little to no expression of SLAMF7 protein in normal tissues, elotuzumab selectively kills multiple myeloma cells with minimal off-target effects [4]. In November 2015, food and drug administration (FDA) approved the combination therapy of elotuzumab with lenalidomide and dexamethasone (Elo-Ld) based on ELOQUENT-2 trial on adult patients with one prior therapy [5]. Elotuzumab combined with pomalidomide and dexamethasone (Elo-Pd) has been approved for use in multiple myeloma patients having received two previous therapies, including lenalidomide and PI (protease inhibitor) in 2018 after ELOQUENT-3 trial [6]. There are numerous side effects associated with elotuzumab combination therapy, including lymphopenia, fatigue, diarrhoea, pyrexia, constipation, cough, peripheral neuropathy and nasopharyngitis [5]. Pulmonary toxicity associated with elotuzumab (combined with lenalidomide/pomalidomide and dexamethasone) was barely described in Phase 1-3 clinical trials [5-7]. Only one case report of elotuzumab induced interstitial lung disease exists [8], with a few cases reports on lenalidomide and pomalidomide induced interstitial lung disease [9-14]. This article covers two cases of acute hypoxic respiratory failure upon administration of elotuzumab, and both treated successfully with corticosteroids.

## Case presentation

Case 1 

A 50-year-old male having a history of multiple myeloma (IgG kappa), on weekly elotuzumab infusion for last five weeks (last infusion four days before admission) along with lenalidomide and dexamethasone presented to emergency with four-day symptoms of shortness of breath, fatigue and malaise. He also reported dry cough. He denied having fever, chills, night sweats, chest pain, abdominal pain, nausea, vomiting, diarrhoea, urinary changes and lower extremity swelling. The patient was diagnosed with pulmonary embolism a month back. Compliance of the patient with therapeutic dose enoxaparin was documented. On initial evaluation, the patient was hypoxic requiring 2-3-litres oxygen, later increased to 15 litres high flow nasal cannula, and eventually stabilized on bilevel positive airway pressure (BiPAP). On physical examination, he had a temperature of 101.3 F, and faint crackles (heard bilaterally at bases) on chest examination. Cardiovascular, gastrointestinal, and neurological examinations were normal.

**Figure 1:**
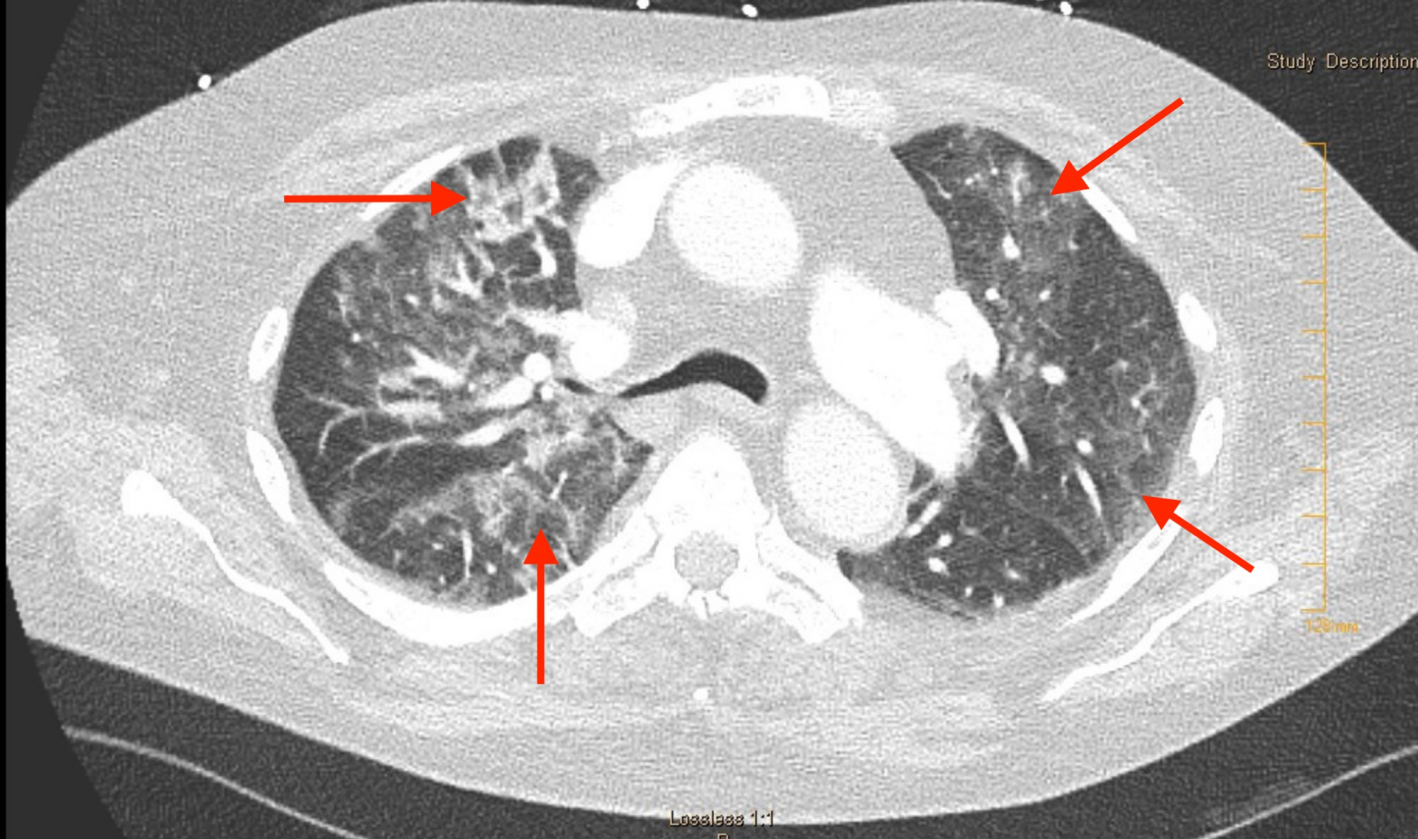
Axial contrast-enhanced CT chest (lung window) showing bilateral diffuse ground-glass opacities.

Laboratory tests revealed thrombocytopenia (platelets (PLT) 16 x 109/L) and anaemia (Hb 7.8 g/dl) consistent with initial baseline values, acute kidney injury (creatinine 1.28 mg/dl), mild pro-BNP elevation (394 pg/ml) with insignificant electrocardiogram abnormality. Viral respiratory pathogens polymerase chain reaction (PCR), COVID-19 PCR, legionella, and streptococcal urine antigens were negative. CT Chest with pulmonary embolism protocol demonstrated the decreased size of previously seen filling defect within right lower lobe segmental artery but new bilateral diffuse ground-glass opacities (Figure 1). Elotuzumab induced pneumonitis, sepsis, pulmonary edema, and multifocal pneumonia were taken as a differential diagnosis. The patient was admitted, blood and urine cultures were sent and started on vancomycin along with meropenem. Normal systolic function (ejection fraction 50%), normal wall motion with no right to left shunt were observed on echocardiogram with bubble study. Continued administration of antibiotics with an observation period of one to two days was suggested by the haematologist along with administration of corticosteroids for possible elotuzumab induced pneumonitis if the patient did not improve under the antibiotics.

**Figure 2:**
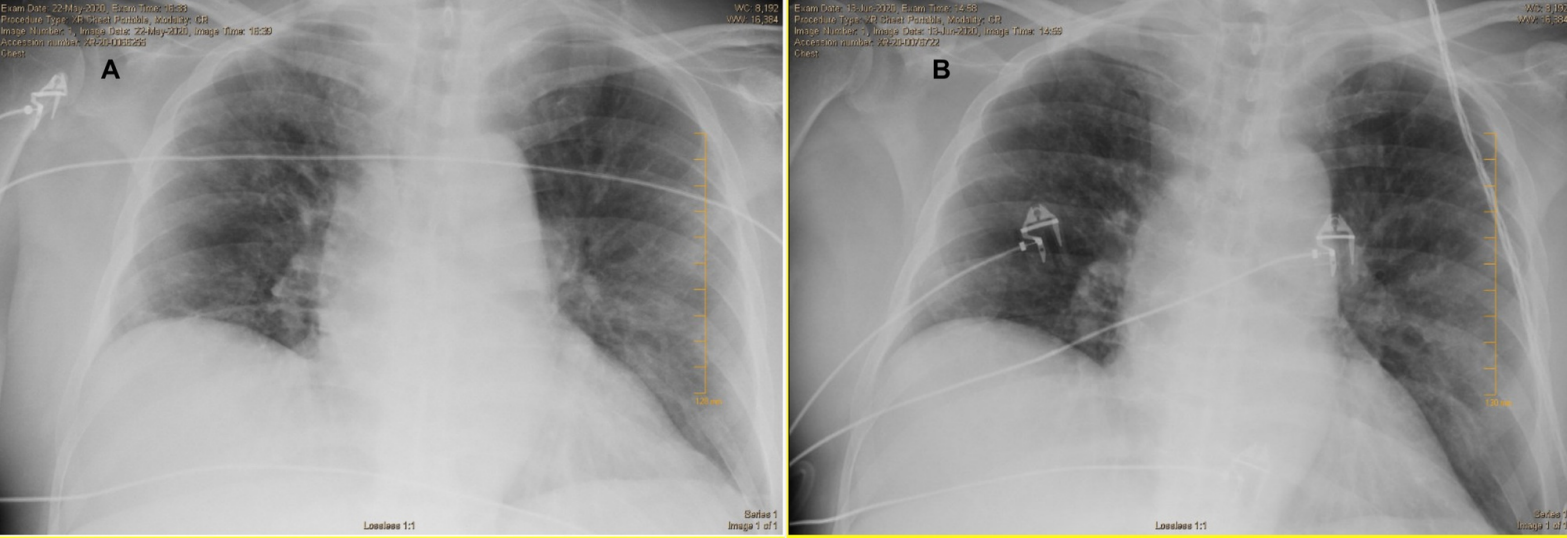
(A) Chest x-ray shows diffuse bilateral interstitial and peripheral opacities. (B) Improved bilateral patchy and hazy airspace opacities.

The patient continued to have shortness of breath requiring 10-15-litres oxygen on high flow nasal cannula and intermittent BIPAP over the next 24 hours. He was started on methylprednisolone (125 mg) every six hours after consultation with the haematologist. This brought down the oxygen requirement to 6 litres/min the next day, but the malaise persisted. Antibiotics were stopped since the urine and blood cultures were negative. Significant improvement was observed on the fourth day of intravenous methylprednisolone as the patient ambulated comfortably. He was not requiring any oxygen, was ambulating without difficulty. The patient was discharged with a prescription of prednisone (40 mg) daily with prolonged taper: 40 mg daily for the first two weeks, 10 mg weekly for next three weeks. Marked improvement of the bilateral opacities was observed after three weeks on chest x-ray (Figure 2). After an additional three weeks, CT chest was ordered, but patient decided to discontinue chemotherapy due to adverse effects, low functional status and opted for hospice care.

Case 2 

A 73-year-old female was admitted with the symptoms of malaise, cough, and shortness of breath on exertion. Past medical history revealed multiple myeloma (stage III light lambda light chain) on weekly elotuzumab for the last nine weeks (last infusion five days ago). Patient endorsed diffuse weakness, chills and sweats for the past couple of days and a 1-day history of cough with progressively worsening shortness of breath. She was uncertain about fever: denied headache, chest pain, abdominal pain, diarrhoea, dysuria. On presentation to the emergency department, the patient was tachycardic and febrile, temperature 101.4 F. Patient was hypoxic and tachypneic while in the emergency department and required 5-liters oxygen by nasal cannula. On examination, she was arousable though lethargic with left-sided chest crackles. 

**Figure 3:**
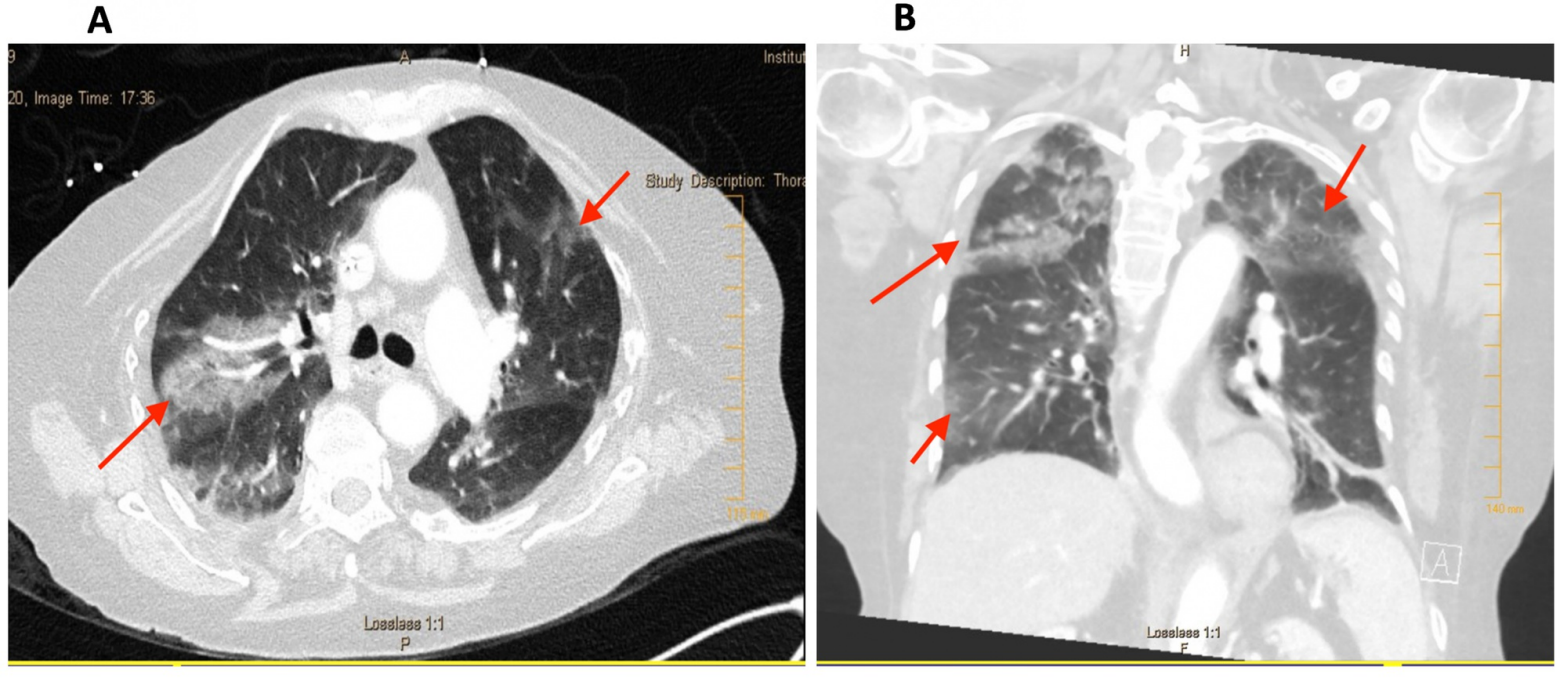
(A) Contrast-enhanced CT chest axial section (lung window) demonstrating patchy bilateral, right greater than left reticular and ground-glass airspace opacities, and (B) similar bilateral ground-glass opacities (coronal view)

Comprehensive metabolic panel and blood tests revealed mild hypokalemia, chronic anaemia, mild acute kidney injury, and lactic acid (0.8). Unremarkable electrocardiogram and negative troponins were documented (pro b-type natriuretic peptide (pro-BNP) 108 pg/ml). Mild interstitial pulmonary edema was observed on chest x-ray. She tested negative for COVID-19. Venous blood gases displayed a pH of 7.3, PCO2 of 41, and PO2 of 39. Blood cultures were sent to the laboratory for evaluation. The patient was admitted to the hospital with the administration of 1.5 L saline bolus and started on vancomycin and piperacillin/tazobactam. Elotuzumab induced pneumonitis, sepsis, pulmonary edema, and pneumonia were considered under differential diagnosis. Corticosteroid administration was recommended by the haematologist if the patient did not improve under the antibiotics during the observation period of one to two days. CT pulmonary angiogram was negative for pulmonary embolism but showed patchy multifocal, right greater than left ground-glass and reticular airspace opacities (Figure 3). Normal systolic function (ejection fraction 65%) and unremarkable regional wall motion were observed on echocardiogram. Respiratory viral pathogen panel by PCR, urine legionella and streptococcus pneumonia antigen were negative.

Patient continued to be tachycardiac and tachypneic, requiring oxygen for the next 72 hours while on antibiotics. Blood cultures, sputum cultures and urine cultures were negative. The patient was started on Prednisone 1mg/kg daily on the fourth day of admission, and the antibiotics were stopped. She was down to 2 litres/min oxygen the next day, and the dyspnea improved. On the third day, marked improvement in the cough was observed as ambulation came close to normal. For the next five days, the patient was continued on prednisone (70 mg) and then discharged with prednisone (60 mg) to wean by 10 mg every five days. CT chest was ordered after completion of steroids, but patient decided to discontinue chemotherapy due to adverse effects and opted for hospice care. Her breathing status remained normal for the next four weeks during follow-ups.

## Discussion

**Figure 4:**
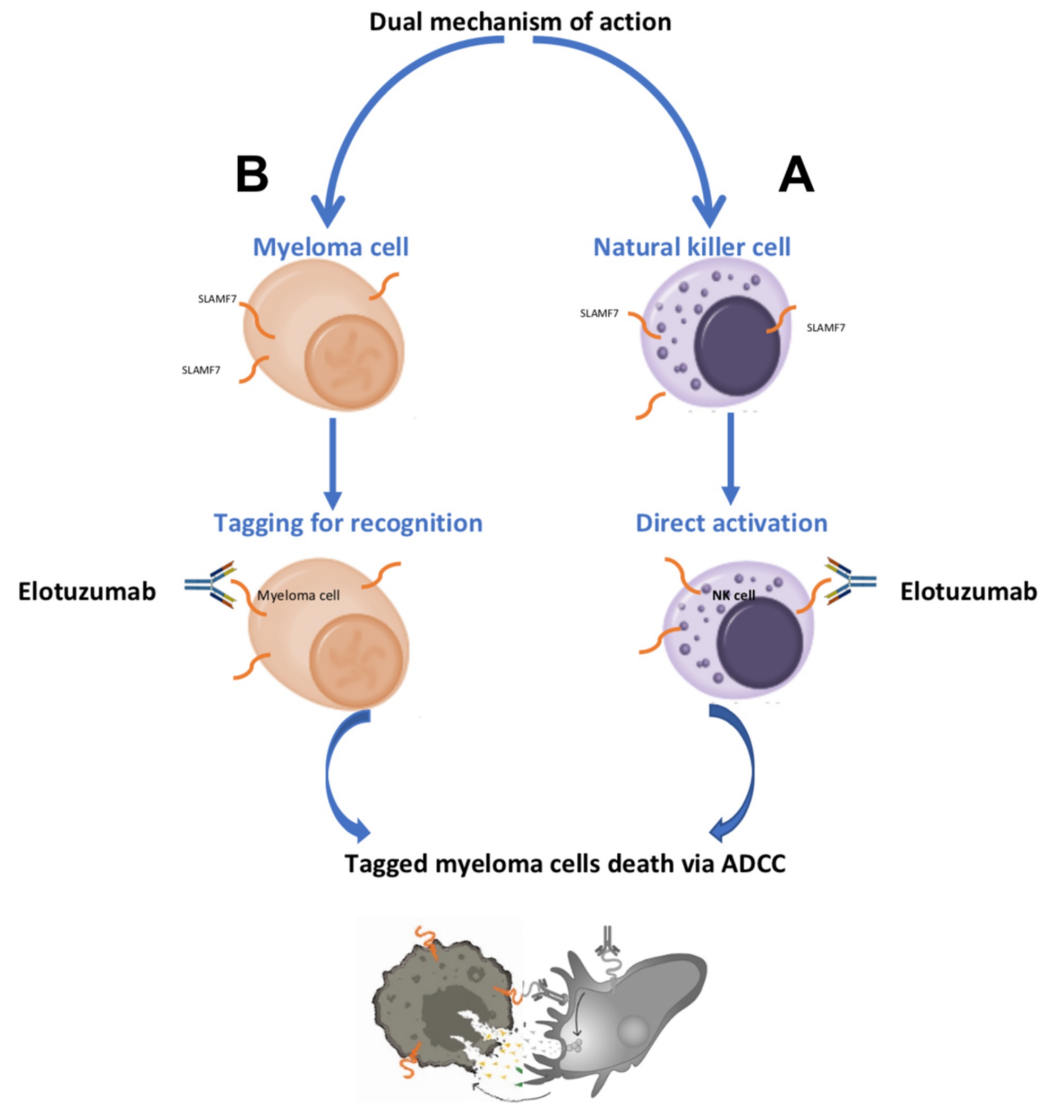
Elotuzumab has dual mechanism of action. (A) Direct activation: elotuzumab binds to SLAMF7 on natural killer cells and activates them. (B) Tagging for recognition: elotuzumab also binds to SLAMF7 on myeloma cells, which tags these myeloma cells for recognition by natural killer cells. Activated natural killer cells recognize myeloma cells that have been tagged by elotuzumab and cell death occurs by antibody-dependent cellular toxicity (ADCC). SLAMF7: Signaling lymphocytic activation molecule-F7, NK cells: Natural killer cells, ADCC: Antibody-dependent cellular toxicity.

Elotuzumab is the first and only immune-stimulatory monoclonal antibody indicated for the combination treatment of patients with multiple myeloma. Mechanism of actions involves targeting SLAMF7. SLAMF7 expression is highest on plasma cells (malignant and normal), natural killer cells, and a subgroup of other immune cells, with no expression on other normal tissue [15]. Elotuzumab has a dual mechanism of actions; it directly activates the immune system, especially natural killer cells by binding to SLAMF7 on their surface. Elotuzumab also targets SLAMF7 on myeloma cells, which tags these myeloma cells for recognition by natural killer cells. Activated natural killer cells recognize myeloma cells that have been tagged by elotuzumab, and cell death occurs by antibody-dependent cellular toxicity (Figure 4).

According to ELOQUENT-2 trial (randomized phase 3 trial evaluating efficacy and safety of elotuzumab in combination with lenalidomide and dexamethasone, as compared with lenalidomide and dexamethasone alone), most common hematological adverse effect with elotuzumab combination therapy was lymphocytopenia [5]. Common non-hematological adverse events were fatigue, diarrhea, cough, peripheral neuropathy, and nasopharyngitis (Table 1). Serious adverse reactions were pneumonia, respiratory tract infections and pyrexia (Table 2).

**Table 1:** Common side effects of E-Ld Arm compared to Ld arm in ELOQUENT-2 trial E-Ld (elotuzumab, lenalidomide and dexamethasone): LD (lenalidomide and dexamethasone): ELOQUENT-2 trial, phase 3 randomized trial evaluating elotuzumab in combination with lenalidomide and dexamethasone compared to lenalidomide and Dexamethasone.

Common adverse Effect	E-Ld Arm	Ld Arm
Lymphocytopenia	77%	49%
Fatigue	62%	52%
Diarrhea	47%	36%
Pyrexia	37%	25%
Constipation	36%	27%
Cough	34%	19%
Peripheral neuropathy	27%	21%
Nasopharyngitis	25%	19%

**Table 2:** Serious side effects of E-Ld Arm compared to Ld arm in ELOQUENT-2 trial E-Ld (elotuzumab, lenalidomide and dexamethasone): LD (lenalidomide and dexamethasone).

Serious adverse effects	E-Ld Arm	Ld Arm
Pneumonia	15%	11%
Pyrexia	7%	5%
Respiratory tract infection	3.1%	1.3%
Pulmonary embolism	3.1%	2.1%
Acute renal failure	2.5%	1.9%

In ELOTUENT-3 trial (randomized trial assessing the efficacy of elotuzumab plus pomalidomide and dexamethasone as compared with pomalidomide and dexamethasone alone in patients with refractory or relapsed and refractory multiple myeloma who had previously received treatment with lenalidomide and a proteasome inhibitor), most common adverse effects were constipation (22% vs 11%) and hyperglycemia (20% vs 15%) in E-Pd (elotuzumab, pomalidomide, dexamethasone) vs Pd (pomalidomide, dexamethasone) arm respectively [6]. Serious adverse reactions were pneumonia and respiratory tract infections (Table 3).

**Table 3:** Serious side effects of E-Pd Arm compared to Pd arm in ELOQUENT-3 trial E-Pd (elotuzumab, pomalidomide, dexamethasone): Pd (pomalidomide, dexamethasone): ELOQUENT-3 trial, phase 3 randomized trial evaluating elotuzumab in combination with pomalidomide and dexamethasone compared to pomalidomide and dexamethasone.

Serious adverse effects	E-Pd Arm	Pd Arm
Pneumonia	13%	11%
Respiratory tract infections	7%	3.6%

As described above, there is an increased risk of respiratory tract infections, including pneumonia with elotuzumab combination treatment. Therefore, exclusion of infection is vital in patients receiving elotuzumab combination therapy and presenting with shortness of breath. Both of our patients presented with fatigue, malaise and shortness of breath. Bilateral pulmonary infiltration was experienced by both patients (five weeks and nine weeks respectively) upon administration of elotuzumab. Both patients demonstrated the appearance of diffuse ground glass and reticular airspace (non-specific but suggestive of drug-induced interstitial pneumonitis) as they developed acute hypoxic respiratory failure. Initially, both patients were started on broad-spectrum antibiotics and blood cultures sent. Respiratory viral pathogen panel by PCR, urine legionella and streptococcus pneumonia antigen were negative. There was no improvement in respiratory status over the next 72 hours while on antibiotics, blood cultures, sputum cultures, and urine cultures were negative. After ruling out infection, we started steroids, and both patients improved rapidly. Bronchoscopy was considered but not done as patients improved significantly while on steroids.

It is also difficult to identify the responsible drug for interstitial lung disease when a patient is receiving combination therapy with elotuzumab, lenalidomide/pomalidomide and dexamethasone. There is ~3.4% incidence of lenalidomide induced ILD at two to eight months [9, 11]. The histological and radiological patterns vary, including organizing/hypersensitivity/(non-specific) interstitial pneumonitis, acute respiratory distress syndrome, and diffuse alveolar haemorrhage. Four prior acute lung toxicity reports exist related to pomalidomide [12-14]. It usually takes 8 - 120 days from initiation of treatment to onset of symptoms. Symptoms of immunomodulatory drugs lung toxicity include cough, dyspnea, fever, and hypoxia; ground-glass opacities being the most common radiological findings [12]. Significant take-away from the first case is that despite his long history of treatment with lenalidomide, interstitial pneumonitis developed acutely after elotuzumab was added to the combined therapy. In the second case presented the patient was receiving weekly elotuzumab with pomalidomide 4 mg daily (day 0-21) but stopped taking pomalidomide after 14 days due to fatigue, weakness, loss of appetite and failure to thrive. There were no respiratory symptoms during pomalidomide treatment and immediately after stopping it. It was after nine weeks of weekly administration of elotuzumab that the patient developed acute hypoxic respiratory failure, and we believe her respiratory failure was secondary to elotuzumab and not pomalidomide.

Tanaka et al. suggest that the mechanism by which elotuzumab induces lung injury (mostly unknown) might be linked to TNF- α secretion, as other studies have revealed monoclonal antibodies (e.g., Rituximab) associated ILD is related to TNF-α secretion [8, 16]. Elotuzumab has been demonstrated to induce TNF- α secretion in vitro [17]. It has also shown a synergistic effect in enhancing TNF- α when combined with lenalidomide. Therefore, interstitial lung disease may develop not only due to enhanced secretion of TNF-α by elotuzumab but also because of the synergistic effect of combining elotuzumab with lenalidomide.

## Conclusions

The administration of elotuzumab can cause interstitial pneumonitis. Presentation is quite similar to an infection; differentiation upon radiology is not conclusive and need full infectious workup. Elotuzumab induced interstitial pneumonitis ought to be in the differential diagnosis in patients on elotuzumab combination therapy and presenting with respiratory distress and hypoxic respiratory failure. Treatment with high dose steroids can be useful and should be started early after ruling out an infection.
